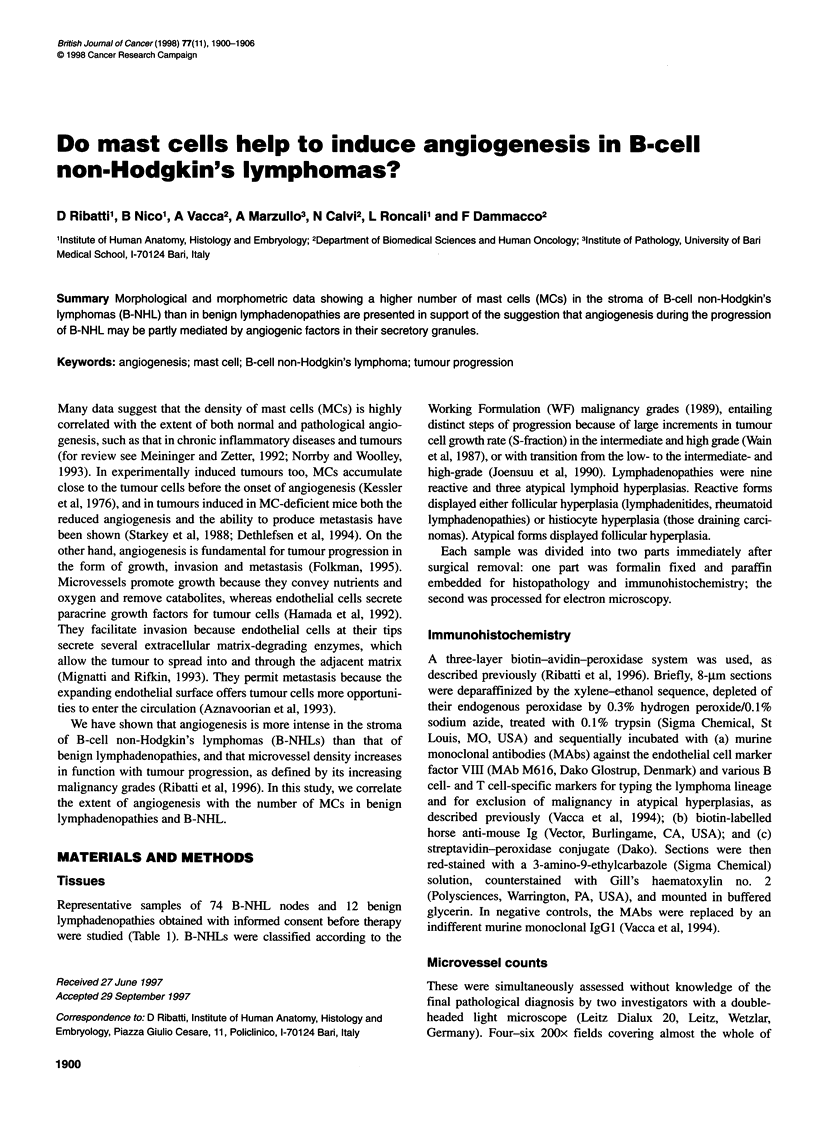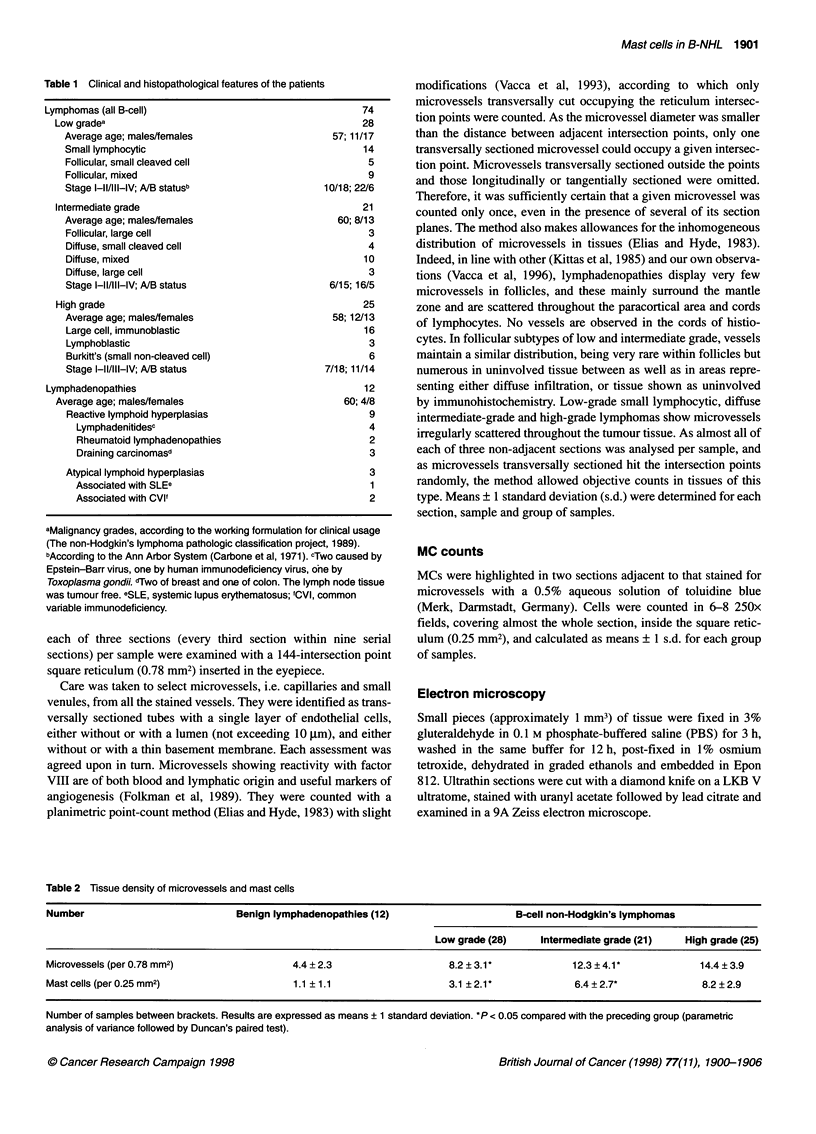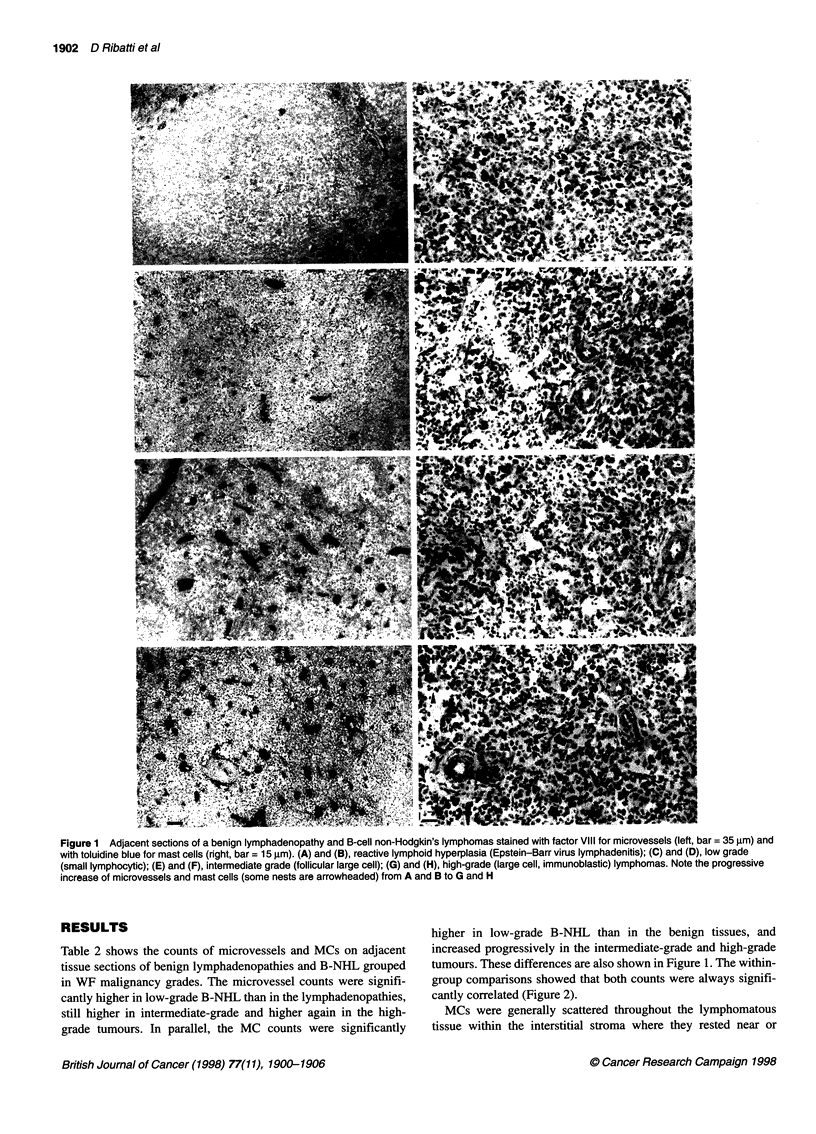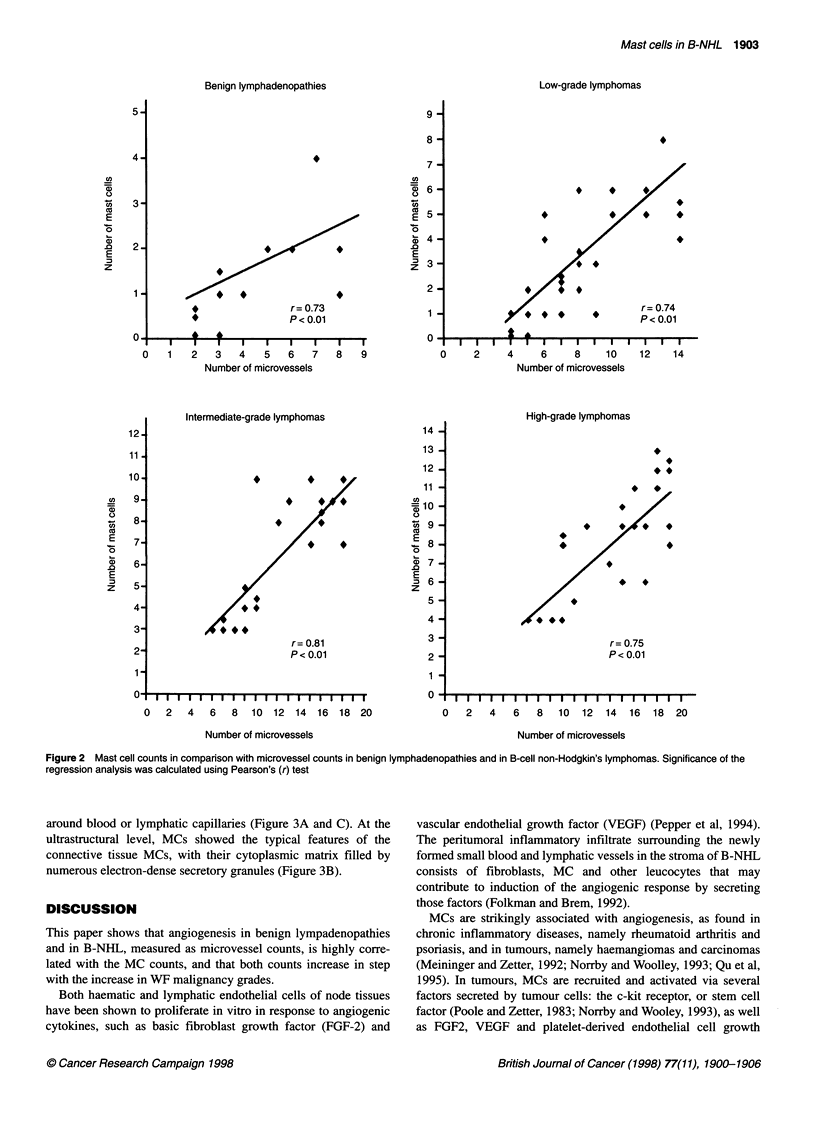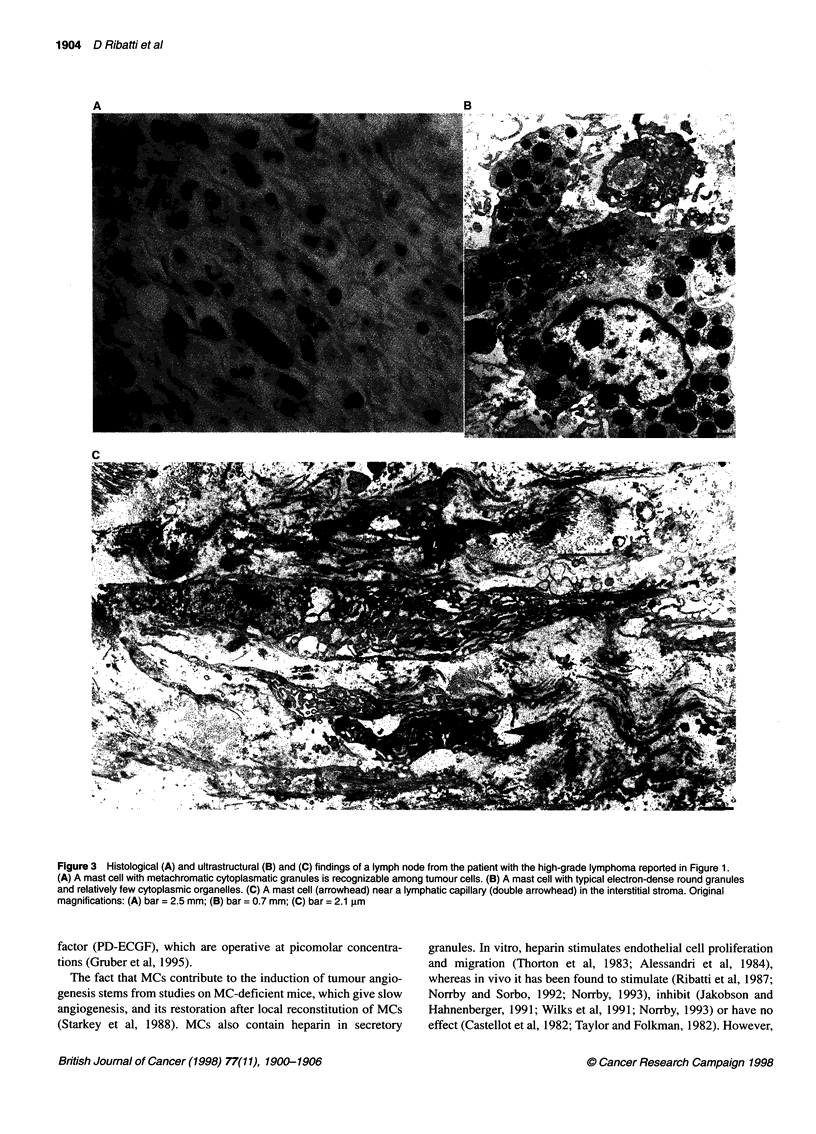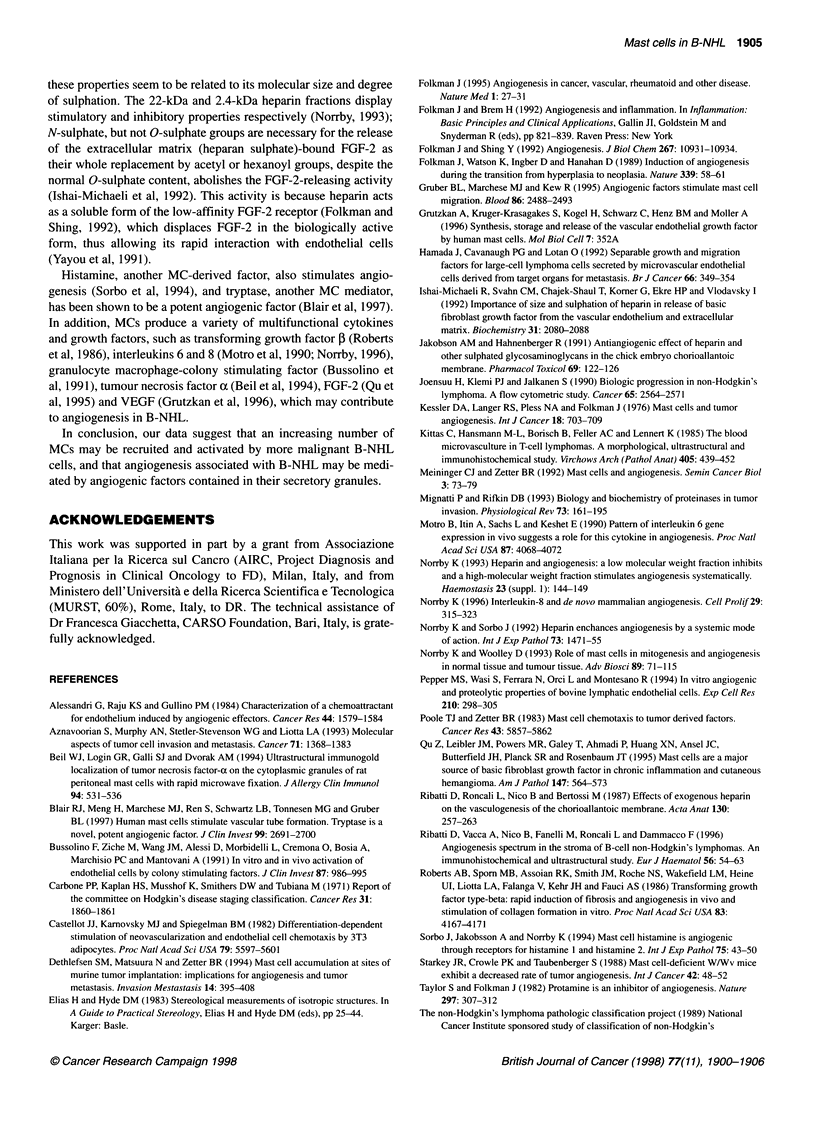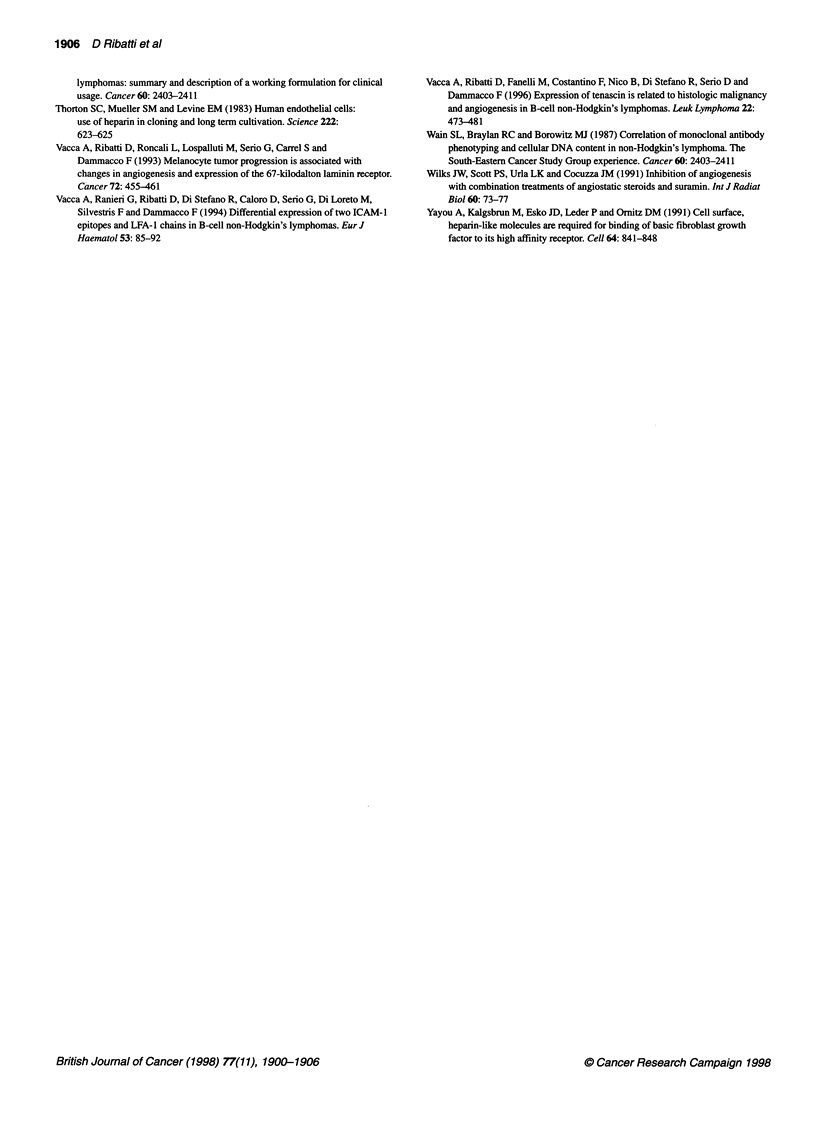# Do mast cells help to induce angiogenesis in B-cell non-Hodgkin's lymphomas?

**DOI:** 10.1038/bjc.1998.316

**Published:** 1998-06

**Authors:** D Ribatti, B Nico, A Vacca, A Marzullo, N Calvi, L Roncali, F Dammacco

**Keywords:** angiogenesis, mast cell, B-cell non-Hodgkin's lymphoma, tumour progression

## Abstract

**Images:**


*British Journal of Cancer* (1998) 77(11), 1900–1906
© 1998 Cancer Research Campaign

### Do mast cells help to induce angiogenesis in B-cell non-Hodgkin's lymphomas?

**D Ribatti[1], B Nico[1], A Vacca[2], A Marzullo[3], N Calvi[2], L Roncali[1] and F Dammacco[2]**

[1]Institute of Human Anatomy, Histology and Embryology; [2]Department of Biomedical Sciences and Human Oncology; [3]Institute of Pathology, University of Bari Medical School, I-70124 Bari, Italy

**Summary** Morphological and morphometric data showing a higher number of mast cells (MCs) in the stroma of B-cell non-Hodgkin's lymphomas (B-NHL) than in benign lymphadenopathies are presented in support of the suggestion that angiogenesis during the progression of B-NHL may be partly mediated by angiogenic factors in their secretory granules.

**Keywords:** angiogenesis; mast cell; B-cell non-Hodgkin's lymphoma; tumour progression

Many data suggest that the density of mast cells (MCs) is highly correlated with the extent of both normal and pathological angiogenesis, such as that in chronic inflammatory diseases and tumours (for review see Meininger and Zetter, 1992; Norrby and Woolley, 1993). In experimentally induced tumours too, MCs accumulate close to the tumour cells before the onset of angiogenesis (Kessler et al, 1976), and in tumours induced in MC-deficient mice both the reduced angiogenesis and the ability to produce metastasis have been shown (Starkey et al, 1988; Dethlefsen et al, 1994). On the other hand, angiogenesis is fundamental for tumour progression in the form of growth, invasion and metastasis (Folkman, 1995). Microvessels promote growth because they convey nutrients and oxygen and remove catabolites, whereas endothelial cells secrete paracrine growth factors for tumour cells (Hamada et al, 1992). They facilitate invasion because endothelial cells at their tips secrete several extracellular matrix-degrading enzymes, which allow the tumour to spread into and through the adjacent matrix (Mignatti and Rifkin, 1993). They permit metastasis because the expanding endothelial surface offers tumour cells more opportunities to enter the circulation (Aznavoorian et al, 1993).

We have shown that angiogenesis is more intense in the stroma of B-cell non-Hodgkin's lymphomas (B-NHLs) than that of benign lymphadenopathies, and that microvessel density increases in function with tumour progression, as defined by its increasing malignancy grades (Ribatti et al, 1996). In this study, we correlate the extent of angiogenesis with the number of MCs in benign lymphadenopathies and B-NHL.

### MATERIALS AND METHODS

### Tissues

Representative samples of 74 B-NHL nodes and 12 benign lymphadenopathies obtained with informed consent before therapy were studied (Table 1). B-NHLs were classified according to the Working Formulation (WF) malignancy grades (1989), entailing distinct steps of progression because of large increments in tumour cell growth rate (S-fraction) in the intermediate and high grade (Wain et al, 1987), or with transition from the low- to the intermediate- and high-grade (Joensuu et al, 1990). Lymphadenopathies were nine reactive and three atypical lymphoid hyperplasias. Reactive forms displayed either follicular hyperplasia (lymphadenitides, rheumatoid lymphadenopathies) or histiocyte hyperplasia (those draining carcinomas). Atypical forms displayed follicular hyperplasia.

Each sample was divided into two parts immediately after surgical removal: one part was formalin fixed and paraffin embedded for histopathology and immunohistochemistry; the second was processed for electron microscopy.

### Immunohistochemistry

A three-layer biotin–avidin–peroxidase system was used, as described previously (Ribatti et al, 1996). Briefly, 8-μm sections were deparaffinized by the xylene–ethanol sequence, depleted of their endogenous peroxidase by 0.3% hydrogen peroxide/0.1% sodium azide, treated with 0.1% trypsin (Sigma Chemical, St Louis, MO, USA) and sequentially incubated with (a) murine monoclonal antibodies (MAbs) against the endothelial cell marker factor VIII (MAb M616, Dako Glostrup, Denmark) and various B cell- and T cell-specific markers for typing the lymphoma lineage and for exclusion of malignancy in atypical hyperplasias, as described previously (Vacca et al, 1994); (b) biotin-labelled horse anti-mouse Ig (Vector, Burlingame, CA, USA); and (c) streptavidin–peroxidase conjugate (Dako). Sections were then red-stained with a 3-amino-9-ethylcarbazole (Sigma Chemical) solution, counterstained with Gill's haematoxylin no. 2 (Polysciences, Warrington, PA, USA), and mounted in buffered glycerin. In negative controls, the MAbs were replaced by an indifferent murine monoclonal IgG1 (Vacca et al, 1994).

### Microvessel counts

These were simultaneously assessed without knowledge of the final pathological diagnosis by two investigators with a double-headed light microscope (Leitz Dialux 20, Leitz, Wetzlar, Germany). Four–six 200× fields covering almost the whole of

*Received 27 June 1997*
*Accepted 29 September 1997*

*Correspondence to:* D Ribatti, Institute of Human Anatomy, Histology and Embryology, Piazza Giulio Cesare, 11, Policlinico, I-70124 Bari, Italy

**Table 1** Clinical and histopathological features of the patients

| | |
|---|---|
| Lymphomas (all B-cell) | 74 |
| Low grade[a] | 28 |
| Average age; males/females | 57; 11/17 |
| Small lymphocytic | 14 |
| Follicular, small cleaved cell | 5 |
| Follicular, mixed | 9 |
| Stage I–II/III–IV; A/B status[b] | 10/18; 22/6 |
| Intermediate grade | 21 |
| Average age; males/females | 60; 8/13 |
| Follicular, large cell | 3 |
| Diffuse, small cleaved cell | 4 |
| Diffuse, mixed | 10 |
| Diffuse, large cell | 3 |
| Stage I–II/III–IV; A/B status | 6/15; 16/5 |
| High grade | 25 |
| Average age; males/females | 58; 12/13 |
| Large cell, immunoblastic | 16 |
| Lymphoblastic | 3 |
| Burkitt's (small non-cleaved cell) | 6 |
| Stage I–II/III–IV; A/B status | 7/18; 11/14 |
| Lymphadenopathies | 12 |
| Average age; males/females | 60; 4/8 |
| Reactive lymphoid hyperplasias | 9 |
| Lymphadenitides[c] | 4 |
| Rheumatoid lymphadenopathies | 2 |
| Draining carcinomas[d] | 3 |
| Atypical lymphoid hyperplasias | 3 |
| Associated with SLE[e] | 1 |
| Associated with CVI[f] | 2 |

[a]Malignancy grades, according to the working formulation for clinical usage (The non-Hodgkin's lymphoma pathologic classification project, 1989). [b]According to the Ann Arbor System (Carbone et al, 1971). [c]Two caused by Epstein–Barr virus, one by human immunodeficiency virus, one by *Toxoplasma gondii*. [d]Two of breast and one of colon. The lymph node tissue was tumour free. [e]SLE, systemic lupus erythematosus; [f]CVI, common variable immunodeficiency.

each of three sections (every third section within nine serial sections) per sample were examined with a 144-intersection point square reticulum (0.78 mm$^2$) inserted in the eyepiece.

Care was taken to select microvessels, i.e. capillaries and small venules, from all the stained vessels. They were identified as transversally sectioned tubes with a single layer of endothelial cells, either without or with a lumen (not exceeding 10 μm), and either without or with a thin basement membrane. Each assessment was agreed upon in turn. Microvessels showing reactivity with factor VIII are of both blood and lymphatic origin and useful markers of angiogenesis (Folkman et al, 1989). They were counted with a planimetric point-count method (Elias and Hyde, 1983) with slight modifications (Vacca et al, 1993), according to which only microvessels transversally cut occupying the reticulum intersection points were counted. As the microvessel diameter was smaller than the distance between adjacent intersection points, only one transversally sectioned microvessel could occupy a given intersection point. Microvessels transversally sectioned outside the points and those longitudinally or tangentially sectioned were omitted. Therefore, it was sufficiently certain that a given microvessel was counted only once, even in the presence of several of its section planes. The method also makes allowances for the inhomogeneous distribution of microvessels in tissues (Elias and Hyde, 1983). Indeed, in line with other (Kittas et al, 1985) and our own observations (Vacca et al, 1996), lymphadenopathies display very few microvessels in follicles, and these mainly surround the mantle zone and are scattered throughout the paracortical area and cords of lymphocytes. No vessels are observed in the cords of histiocytes. In follicular subtypes of low and intermediate grade, vessels maintain a similar distribution, being very rare within follicles but numerous in uninvolved tissue between as well as in areas representing either diffuse infiltration, or tissue shown as uninvolved by immunohistochemistry. Low-grade small lymphocytic, diffuse intermediate-grade and high-grade lymphomas show microvessels irregularly scattered throughout the tumour tissue. As almost all of each of three non-adjacent sections was analysed per sample, and as microvessels transversally sectioned hit the intersection points randomly, the method allowed objective counts in tissues of this type. Means ± 1 standard deviation (s.d.) were determined for each section, sample and group of samples.

### MC counts

MCs were highlighted in two sections adjacent to that stained for microvessels with a 0.5% aqueous solution of toluidine blue (Merk, Darmstadt, Germany). Cells were counted in 6–8 250× fields, covering almost the whole section, inside the square reticulum (0.25 mm$^2$), and calculated as means ± 1 s.d. for each group of samples.

### Electron microscopy

Small pieces (approximately 1 mm$^3$) of tissue were fixed in 3% gluteraldehyde in 0.1 M phosphate-buffered saline (PBS) for 3 h, washed in the same buffer for 12 h, post-fixed in 1% osmium tetroxide, dehydrated in graded ethanols and embedded in Epon 812. Ultrathin sections were cut with a diamond knife on a LKB V ultratome, stained with uranyl acetate followed by lead citrate and examined in a 9A Zeiss electron microscope.

**Table 2** Tissue density of microvessels and mast cells

| Number | Benign lymphadenopathies (12) | B-cell non-Hodgkin's lymphomas | | |
|---|---|---|---|---|
| | | Low grade (28) | Intermediate grade (21) | High grade (25) |
| Microvessels (per 0.78 mm$^2$) | 4.4 ± 2.3 | 8.2 ± 3.1* | 12.3 ± 4.1* | 14.4 ± 3.9 |
| Mast cells (per 0.25 mm$^2$) | 1.1 ± 1.1 | 3.1 ± 2.1* | 6.4 ± 2.7* | 8.2 ± 2.9 |

Number of samples between brackets. Results are expressed as means ± 1 standard deviation. *$P < 0.05$ compared with the preceding group (parametric analysis of variance followed by Duncan's paired test).

© Cancer Research Campaign 1998

**Figure 1** Adjacent sections of a benign lymphadenopathy and B-cell non-Hodgkin's lymphomas stained with factor VIII for microvessels (left, bar = 35 μm) and with toluidine blue for mast cells (right, bar = 15 μm). (**A**) and (**B**), reactive lymphoid hyperplasia (Epstein–Barr virus lymphadenitis); (**C**) and (**D**), low grade (small lymphocytic); (**E**) and (**F**), intermediate grade (follicular large cell); (**G**) and (**H**), high-grade (large cell, immunoblastic) lymphomas. Note the progressive increase of microvessels and mast cells (some nests are arrowheaded) from **A** and **B** to **G** and **H**

### RESULTS

Table 2 shows the counts of microvessels and MCs on adjacent tissue sections of benign lymphadenopathies and B-NHL grouped in WF malignancy grades. The microvessel counts were significantly higher in low-grade B-NHL than in the lymphadenopathies, still higher in intermediate-grade and higher again in the high-grade tumours. In parallel, the MC counts were significantly higher in low-grade B-NHL than in the benign tissues, and increased progressively in the intermediate-grade and high-grade tumours. These differences are also shown in Figure 1. The within-group comparisons showed that both counts were always significantly correlated (Figure 2).

MCs were generally scattered throughout the lymphomatous tissue within the interstitial stroma where they rested near or

© Cancer Research Campaign 1998

**Figure 2** Mast cell counts in comparison with microvessel counts in benign lymphadenopathies and in B-cell non-Hodgkin's lymphomas. Significance of the regression analysis was calculated using Pearson's (*r*) test around blood or lymphatic capillaries (Figure 3A and C). At the ultrastructural level, MCs showed the typical features of the connective tissue MCs, with their cytoplasmic matrix filled by numerous electron-dense secretory granules (Figure 3B).

### DISCUSSION

This paper shows that angiogenesis in benign lympadenopathies and in B-NHL, measured as microvessel counts, is highly correlated with the MC counts, and that both counts increase in step with the increase in WF malignancy grades.

Both haematic and lymphatic endothelial cells of node tissues have been shown to proliferate in vitro in response to angiogenic cytokines, such as basic fibroblast growth factor (FGF-2) and vascular endothelial growth factor (VEGF) (Pepper et al, 1994). The peritumoral inflammatory infiltrate surrounding the newly formed small blood and lymphatic vessels in the stroma of B-NHL consists of fibroblasts, MC and other leucocytes that may contribute to induction of the angiogenic response by secreting those factors (Folkman and Brem, 1992).

MCs are strikingly associated with angiogenesis, as found in chronic inflammatory diseases, namely rheumatoid arthritis and psoriasis, and in tumours, namely haemangiomas and carcinomas (Meininger and Zetter, 1992; Norrby and Woolley, 1993; Qu et al, 1995). In tumours, MCs are recruited and activated via several factors secreted by tumour cells: the c-kit receptor, or stem cell factor (Poole and Zetter, 1983; Norrby and Wooley, 1993), as well as FGF2, VEGF and platelet-derived endothelial cell growth

**Figure 3** Histological (**A**) and ultrastructural (**B**) and (**C**) findings of a lymph node from the patient with the high-grade lymphoma reported in Figure 1. (**A**) A mast cell with metachromatic cytoplasmatic granules is recognizable among tumour cells. (**B**) A mast cell with typical electron-dense round granules and relatively few cytoplasmic organelles. (**C**) A mast cell (arrowhead) near a lymphatic capillary (double arrowhead) in the interstitial stroma. Original magnifications: (**A**) bar = 2.5 mm; (**B**) bar = 0.7 mm; (**C**) bar = 2.1 μm factor (PD-ECGF), which are operative at picomolar concentrations (Gruber et al, 1995).

The fact that MCs contribute to the induction of tumour angiogenesis stems from studies on MC-deficient mice, which give slow angiogenesis, and its restoration after local reconstitution of MCs (Starkey et al, 1988). MCs also contain heparin in secretory granules. In vitro, heparin stimulates endothelial cell proliferation and migration (Thorton et al, 1983; Alessandri et al, 1984), whereas in vivo it has been found to stimulate (Ribatti et al, 1987; Norrby and Sorbo, 1992; Norrby, 1993), inhibit (Jakobson and Hahnenberger, 1991; Wilks et al, 1991; Norrby, 1993) or have no effect (Castellot et al, 1982; Taylor and Folkman, 1982). However,

*© Cancer Research Campaign 1998*

these properties seem to be related to its molecular size and degree of sulphation. The 22-kDa and 2.4-kDa heparin fractions display stimulatory and inhibitory properties respectively (Norrby, 1993); *N*-sulphate, but not *O*-sulphate groups are necessary for the release of the extracellular matrix (heparan sulphate)-bound FGF-2 as their whole replacement by acetyl or hexanoyl groups, despite the normal *O*-sulphate content, abolishes the FGF-2-releasing activity (Ishai-Michaeli et al, 1992). This activity is because heparin acts as a soluble form of the low-affinity FGF-2 receptor (Folkman and Shing, 1992), which displaces FGF-2 in the biologically active form, thus allowing its rapid interaction with endothelial cells (Yayou et al, 1991).

Histamine, another MC-derived factor, also stimulates angiogenesis (Sorbo et al, 1994), and tryptase, another MC mediator, has been shown to be a potent angiogenic factor (Blair et al, 1997). In addition, MCs produce a variety of multifunctional cytokines and growth factors, such as transforming growth factor β (Roberts et al, 1986), interleukins 6 and 8 (Motro et al, 1990; Norrby, 1996), granulocyte macrophage-colony stimulating factor (Bussolino et al, 1991), tumour necrosis factor α (Beil et al, 1994), FGF-2 (Qu et al, 1995) and VEGF (Grutzkan et al, 1996), which may contribute to angiogenesis in B-NHL.

In conclusion, our data suggest that an increasing number of MCs may be recruited and activated by more malignant B-NHL cells, and that angiogenesis associated with B-NHL may be mediated by angiogenic factors contained in their secretory granules.

### ACKNOWLEDGEMENTS

This work was supported in part by a grant from Associazione Italiana per la Ricerca sul Cancro (AIRC, Project Diagnosis and Prognosis in Clinical Oncology to FD), Milan, Italy, and from Ministero dell'Università e della Ricerca Scientifica e Tecnologica (MURST, 60%), Rome, Italy, to DR. The technical assistance of Dr Francesca Giacchetta, CARSO Foundation, Bari, Italy, is gratefully acknowledged.

### REFERENCES

Alessandri G, Raju KS and Gullino PM (1984) Characterization of a chemoattractant for endothelium induced by angiogenic effectors. *Cancer Res* **44**: 1579–1584

Aznavoorian S, Murphy AN, Stetler-Stevenson WG and Liotta LA (1993) Molecular aspects of tumor cell invasion and metastasis. *Cancer* **71**: 1368–1383

Beil WJ, Login GR, Galli SJ and Dvorak AM (1994) Ultrastructural immunogold localization of tumor necrosis factor-α on the cytoplasmic granules of rat peritoneal mast cells with rapid microwave fixation. *J Allergy Clin Immunol* **94**: 531–536

Blair RJ, Meng H, Marchese MJ, Ren S, Schwartz LB, Tonnesen MG and Gruber BL (1997) Human mast cells stimulate vascular tube formation. Tryptase is a novel, potent angiogenic factor. *J Clin Invest* **99**: 2691–2700

Bussolino F, Ziche M, Wang JM, Alessi D, Morbidelli L, Cremona O, Bosia A, Marchisio PC and Mantovani A (1991) In vitro and in vivo activation of endothelial cells by colony stimulating factors. *J Clin Invest* **87**: 986–995

Carbone PP, Kaplan HS, Musshof K, Smithers DW and Tubiana M (1971) Report of the committee on Hodgkin's disease staging classification. *Cancer Res* **31**: 1860–1861

Castellot JJ, Karnovsky MJ and Spiegelman BM (1982) Differentiation-dependent stimulation of neovascularization and endothelial cell chemotaxis by 3T3 adipocytes. *Proc Natl Acad Sci USA* **79**: 5597–5601

Dethlefsen SM, Matsuura N and Zetter BR (1994) Mast cell accumulation at sites of murine tumor implantation: implications for angiogenesis and tumor metastasis. *Invasion Mestastasis* **14**: 395–408

Elias H and Hyde DM (1983) Stereological measurements of isotropic structures. In *A Guide to Practical Stereology*, Elias H and Hyde DM (eds), pp 25–44. Karger: Basle.

Folkman J (1995) Angiogenesis in cancer, vascular, rheumatoid and other disease. *Nature Med* **1**: 27–31

Folkman J and Brem H (1992) Angiogenesis and inflammation. In *Inflammation: Basic Principles and Clinical Applications*, Gallin JI, Goldstein M and Snyderman R (eds), pp 821–839. Raven Press: New York

Folkman J and Shing Y (1992) Angiogenesis. *J Biol Chem* **267**: 10931–10934.

Folkman J, Watson K, Ingber D and Hanahan D (1989) Induction of angiogenesis during the transition from hyperplasia to neoplasia. *Nature* **339**: 58–61

Gruber BL, Marchese MJ and Kew R (1995) Angiogenic factors stimulate mast cell migration. *Blood* **86**: 2488–2493

Grutzkan A, Kruger-Krasagakes S, Kogel H, Schwarz C, Henz BM and Moller A (1996) Synthesis, storage and release of the vascular endothelial growth factor by human mast cells. *Mol Biol Cell* **7**: 352A

Hamada J, Cavanaugh PG and Lotan O (1992) Separable growth and migration factors for large-cell lymphoma cells secreted by microvascular endothelial cells derived from target organs for metastasis. *Br J Cancer* **66**: 349–354

Ishai-Michaeli R, Svahn CM, Chajek-Shaul T, Korner G, Ekre HP and Vlodavsky I (1992) Importance of size and sulphation of heparin in release of basic fibroblast growth factor from the vascular endothelium and extracellular matrix. *Biochemistry* **31**: 2080–2088

Jakobson AM and Hahnenberger R (1991) Antiangiogenic effect of heparin and other sulphated glycosaminoglycans in the chick embryo chorioallantoic membrane. *Pharmacol Toxicol* **69**: 122–126

Joensuu H, Klemi PJ and Jalkanen S (1990) Biologic progression in non-Hodgkin's lymphoma. A flow cytometric study. *Cancer* **65**: 2564–2571

Kessler DA, Langer RS, Pless NA and Folkman J (1976) Mast cells and tumor angiogenesis. *Int J Cancer* **18**: 703–709

Kittas C, Hansmann M-L, Borisch B, Feller AC and Lennert K (1985) The blood microvasculture in T-cell lymphomas. A morphological, ultrastructural and immunohistochemical study. *Virchows Arch (Pathol Anat)* **405**: 439–452

Meininger CJ and Zetter BR (1992) Mast cells and angiogenesis. *Semin Cancer Biol* **3**: 73–79

Mignatti P and Rifkin DB (1993) Biology and biochemistry of proteinases in tumor invasion. *Physiological Rev* **73**: 161–195

Motro B, Itin A, Sachs L and Keshet E (1990) Pattern of interleukin 6 gene expression in vivo suggests a role for this cytokine in angiogenesis. *Proc Natl Acad Sci USA* **87**: 4068–4072

Norrby K (1993) Heparin and angiogenesis: a low molecular weight fraction inhibits and a high-molecular weight fraction stimulates angiogenesis systematically. *Haemostasis* **23** (suppl. 1): 144–149

Norrby K (1996) Interleukin-8 and *de novo* mammalian angiogenesis. *Cell Prolif* **29**: 315–323

Norrby K and Sorbo J (1992) Heparin enchances angiogenesis by a systemic mode of action. *Int J Exp Pathol* **73**: 1471–55

Norrby K and Woolley D (1993) Role of mast cells in mitogenesis and angiogenesis in normal tissue and tumour tissue. *Adv Biosci* **89**: 71–115

Pepper MS, Wasi S, Ferrara N, Orci L and Montesano R (1994) In vitro angiogenic and proteolytic properties of bovine lymphatic endothelial cells. *Exp Cell Res* **210**: 298–305

Poole TJ and Zetter BR (1983) Mast cell chemotaxis to tumor derived factors. *Cancer Res* **43**: 5857–5862

Qu Z, Leibler JM, Powers MR, Galey T, Ahmadi P, Huang XN, Ansel JC, Butterfield JH, Planck SR and Rosenbaum JT (1995) Mast cells are a major source of basic fibroblast growth factor in chronic inflammation and cutaneous hemangioma. *Am J Pathol* **147**: 564–573

Ribatti D, Roncali L, Nico B and Bertossi M (1987) Effects of exogenous heparin on the vasculogenesis of the chorioallantoic membrane. *Acta Anat* **130**: 257–263

Ribatti D, Vacca A, Nico B, Fanelli M, Roncali L and Dammacco F (1996) Angiogenesis spectrum in the stroma of B-cell non-Hodgkin's lymphomas. An immunohistochemical and ultrastructural study. *Eur J Haematol* **56**: 54–63

Roberts AB, Sporn MB, Assoian RK, Smith JM, Roche NS, Wakefield LM, Heine UI, Liotta LA, Falanga V, Kehr JH and Fauci AS (1986) Transforming growth factor type-beta: rapid induction of fibrosis and angiogenesis in vivo and stimulation of collagen formation in vitro. *Proc Natl Acad Sci USA* **83**: 4167–4171

Sorbo J, Jakobsson A and Norrby K (1994) Mast cell histamine is angiogenic through receptors for histamine 1 and histamine 2. *Int J Exp Pathol* **75**: 43–50

Starkey JR, Crowle PK and Taubenberger S (1988) Mast cell-deficient W/Wv mice exhibit a decreased rate of tumor angiogenesis. *Int J Cancer* **42**: 48–52

Taylor S and Folkman J (1982) Protamine is an inhibitor of angiogenesis. *Nature* **297**: 307–312

The non-Hodgkin's lymphoma pathologic classification project (1989) National Cancer Institute sponsored study of classification of non-Hodgkin's

© Cancer Research Campaign 1998

lymphomas: summary and description of a working formulation for clinical usage. *Cancer* **60**: 2403–2411

Thorton SC, Mueller SM and Levine EM (1983) Human endothelial cells: use of heparin in cloning and long term cultivation. *Science* **222**: 623–625

Vacca A, Ribatti D, Roncali L, Lospalluti M, Serio G, Carrel S and Dammacco F (1993) Melanocyte tumor progression is associated with changes in angiogenesis and expression of the 67-kilodalton laminin receptor. *Cancer* **72**: 455–461

Vacca A, Ranieri G, Ribatti D, Di Stefano R, Caloro D, Serio G, Di Loreto M, Silvestris F and Dammacco F (1994) Differential expression of two ICAM-1 epitopes and LFA-1 chains in B-cell non-Hodgkin's lymphomas. *Eur J Haematol* **53**: 85–92

Vacca A, Ribatti D, Fanelli M, Costantino F, Nico B, Di Stefano R, Serio D and Dammacco F (1996) Expression of tenascin is related to histologic malignancy and angiogenesis in B-cell non-Hodgkin's lymphomas. *Leuk Lymphoma* **22**: 473–481

Wain SL, Braylan RC and Borowitz MJ (1987) Correlation of monoclonal antibody phenotyping and cellular DNA content in non-Hodgkin's lymphoma. The South-Eastern Cancer Study Group experience. *Cancer* **60**: 2403–2411

Wilks JW, Scott PS, Urla LK and Cocuzza JM (1991) Inhibition of angiogenesis with combination treatments of angiostatic steroids and suramin. *Int J Radiat Biol* **60**: 73–77

Yayou A, Kalgsbrun M, Esko JD, Leder P and Ornitz DM (1991) Cell surface, heparin-like molecules are required for binding of basic fibroblast growth factor to its high affinity receptor. *Cell* **64**: 841–848

*© Cancer Research Campaign 1998*